# Condylar volume and surface in Caucasian young adult subjects

**DOI:** 10.1186/1471-2342-10-28

**Published:** 2010-12-31

**Authors:** Simona Tecco, Matteo Saccucci, Riccardo Nucera, Antonella Polimeni, Mario Pagnoni, Giancarlo Cordasco, Felice Festa, Giorgio Iannetti

**Affiliations:** 1 Department of Oral Science, Nano and Biotechnology, University G.D'Annunzio, Via dei Vestini 31, Chieti, 66013, Italy; 2 Department of Head and Neck Pathology, University La Sapienza, Rome, Italy; 3 Department of Oral Science, University of Messina, Italy

## Abstract

**Background:**

There have been no quantitative standards for volumetric and surface measurements of the mandibular condyle in Caucasian population. However, the recently developed cone-beam computed tomography (CBCT) system allows measurement of these parameters with high accuracy.

**Methods:**

CBCT was used to measure the condylar volume, surface and the volume to surface ratio, called the Morphometric Index (MI), of 300 temporo-mandibular joints (TMJ) in 150 Caucasian young adult subjects, with varied malocclusions, without pain or dysfunction of TMJs.

**Results:**

The condylar volume was 691.26 ± 54.52 mm^3 ^in males and 669.65 ± 58.80 mm^3 ^in, and was significantly higher (*p*< 0.001) in the males. The same was observed for the condylar surface, although without statistical significance (406.02 ± 55.22 mm^2 ^in males and 394.77 ± 60.73 mm^2 ^in females).

Furthermore, the condylar volume (693.61 ± 62.82 mm^3 ^) in the right TMJ was significantly higher than in the left (666.99 ± 48.67 mm^3^, *p *< 0.001) as was the condylar surface (411.24 ± 57.99 mm^2 ^in the right TMJ and 389.41 ± 56.63 mm^2 ^in the left TMJ; *t *= 3.29; *p *< 0.01). The MI is 1.72 ± 0.17 for the whole sample, with no significant difference between males and females or the right and left sides.

**Conclusion:**

These data from temporomandibular joints of patients without pain or clinical dysfunction might serve as examples of normal TMJ's in the general population not seeking orthodontic care.

## Background

Three-dimensional (3-D) Cone Beam Computed Tomography (CBCT) systems [1] produce images with high resolution (100-300 micron) and minimal distortion, that allows evaluating osteoarthrosis in the temporomandibular joint (TMJ). It also allows for correlating the diagnosis to the age of the patient, [2,3] individuation of remodeling areas, [4] erosions, osteophytes, lines of fracture, bone resorption, [1] condylar displacement after orthognatic surgery, [5] or particular clinical evidences, such as the trifid condyle [6].

CBCT provides a method to calculate the volume of the structure, which could be interesting from research and clinical points of view.

First, from clinical point of view, the multiple image views and computer-generated 3-D models of patients could allow the diagnostician to better visualize the potential therapeutic effects before they are actually rendered in a "spatial plane concept," which is perceived as the next progressive step in dental imaging.

The 3-D evaluation of the mandibular condyle could be particularly interesting, because the condyle, the primary center of growth in the mandible, responds to the continuous stimuli through a remodeling process, and thus plays an important role in the final (adult) dimension of the mandible. Hence, its volume can be related to the mandibular final dimension as well as to the relationship between maxillary and mandibular bases.

During young adulthood, the condyle (its shape and volume) demonstrates adaptability to functional stimuli and this adaption can play an important role in the stability of long-term orthodontic and orthognathic therapies [7].

Also, during adulthood, owing to its adaptability, the condyle is often subjected to remodeling processes (i.e., flattening, erosion, sclerosis, osteophytes, and resorption), which could affect its volume and shape [3]. Such changes are more associated with (1) arthritis, which can affect the condylar volume, and the asymmetry as recently assessed in humans, [8] and (2) anterior displacement of the disc as demonstrated in the rabbit joints [9] - which has been shown to cause an increase in the condylar volume, probably because of hyperplasia of condylar cartilage and increase in nonarticulating surface of the condylar head.

The purpose of this study was to determine the condylar volume and surface area in young adult Caucasian subjects, through CBCT images, without pain or dysfunctions in their TMJs. The data derived from this study might provide a baseline for better understanding of the relationship between condylar volume and surface, called the Morphometric Index (MI), to define the altered shape of the condyles.

## Methods

### The sample

The 3-D CBCT scans of 300 temporomandibular joints (TMJ) in 150 Caucasian young adults subjects (mean age 19.2 years; range: 15-29; 74 males and 76 females), who did not show pain or dysfunction of TMJ, and referred to the public clinic of head and facial medicine for orthodontic evaluation, were examined. The selection of subjects was made among those who did not show pain or dysfunction of TMJ [10] at the first orthodontic visit.

From a clinical point of view, we evaluated whether there was spontaneously evoked pain, localized in the muscles of mastication, the preauricular area, and/or the TMJs, which may be related to trauma (such as a blow to the face), interanal derangement, inflammatory or degenerative arthritis, or by the mandible being pushed back towards the ears whenever the patient chews or swallows. The muscles around the TMJ used for chewing were palpated, and head and neck pain were evaluated. In addition, difficulty opening the mouth normally was evaluated. Then, earache, headache, and facial pain were evaluated. Finally, limited or asymmetric jaw movement and joint sounds (i.e., clicking, popping, grating, crepitus) in the temporomandibular joint were evaluated to exclude TMJ dysfunction signs.

The CBCT scans were taken as orthodontic diagnostic tools. In our orthodontic department, patients who come for evaluation are asked to take the traditional radiographs (i.e., lateral skull radiographs, postero-anterior skull radiographs, and orthopantomographs) or, alternatively, to make a CBCT acquisition, avoiding the three radiographs. The sample included in this retrospective study was selected among those patients who chose to have CBCT scans.

The protocol was evaluated by the Ethical Committee of the University of Chiati, that stated no ethical approval of the protocol is necessary for this retrospective study, as patients have gone spontaneously to the Department for their medical evaluation of the orthodontic problem. Only a signed informed agreement was obtained from each subject, by the radiological study, to know and approve the medical procedure.

The evaluation of CBCT data included:

1. Condylar volume

2. Condylar surface

### Condylar 3D reconstruction, and calculation of volume and surface

Cone Beam Computerized Tomography (CBCT) data sets were acquired using ILUMA™ (IMTEC, 3 M Company, Ardmore, Oklahoma, USA), with a reconstructed layer thickness of 0.1 mm and a 512 × 512 matrix. The device was operated at 120 kVp and 3-8 mA using a high-frequency generator with a fixed anode and a 0.5-mm focal spot. A single 40-s (because the complete volume of the head was taken) high-resolution scan of each skull was made with a voxel size (mm^3^) set at 0.25 mm^3^, and a 17.0 mm diameter and 13.2 cm field of view.

All CBCT images were taken with the subjects sitting in an upright position, with their backs as nearly perpendicular to the floor as possible. The head was always stabilized with ear rods in the external auditory meatus. The subjects were instructed to look into their own eyes in a mirror 1,5 m in front of them to obtain the natural head position.

**Figure 1:**
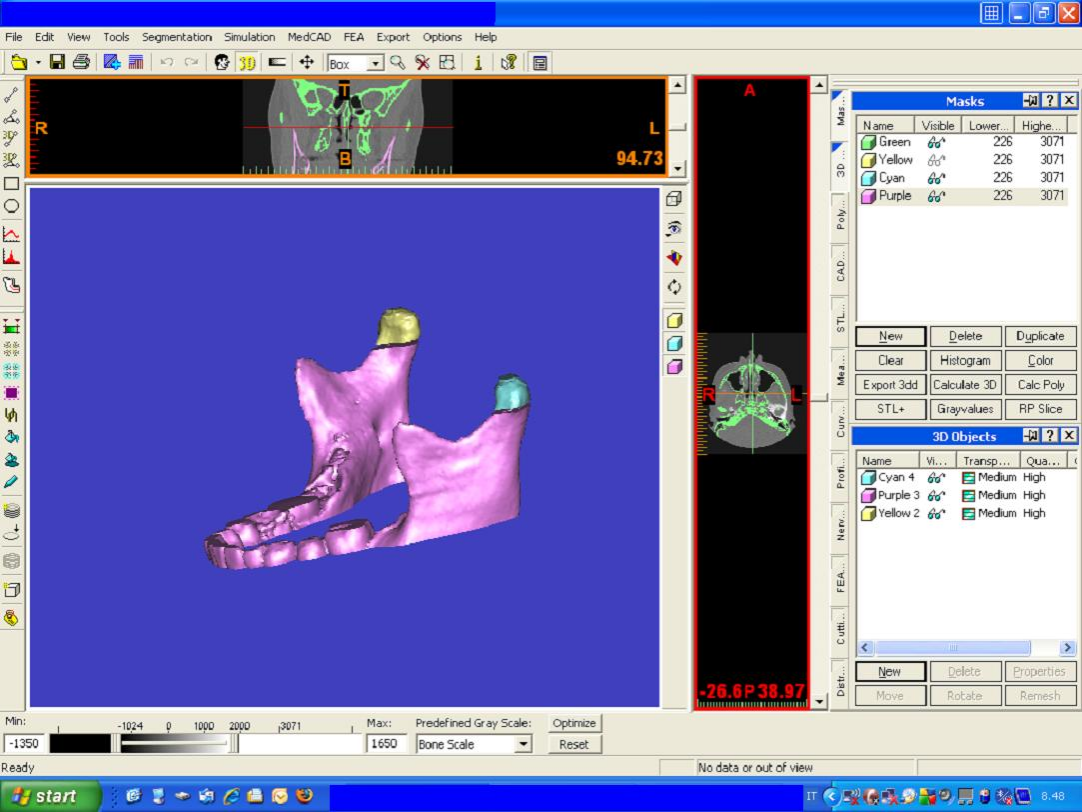
**Condylar 3-D reconstructions with Mimics software**.

Image segmentation of the anatomic structures of interest based on 2-D Digital Imaging and Communications in Medicine (DICOM) formatted data provided different planes of view as well as three-dimensionally reconstructed volumes using Mimics™ 9.0 software (Materialise NV Technologielaan, Leuven, Belgium) (Figure 1).

The 3-D reconstruction of the condyle requires the mandibular condyle to be separated in all the three planes of space from all the other structures, mostly the soft tissues.

Each condyle was visualized in the recommended range of bone density (range of gray scale from -1350 to 1650) and segmented using an adaptive threshold, which was visually checked prior to making 3-D and volumetric measurements.

**Figure 2:**
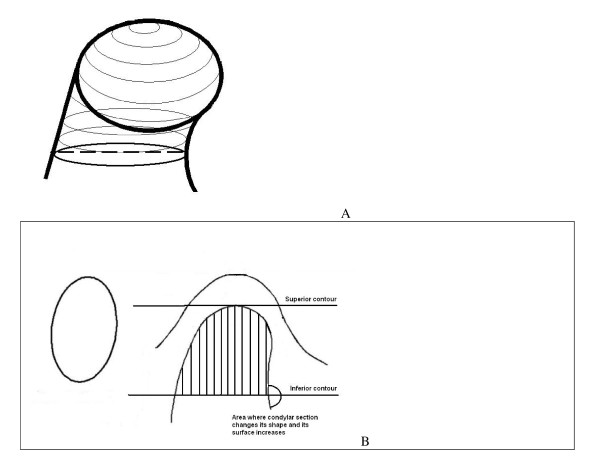
**(a-b) Limits of condylar reconstructions**. (a) Condylar transversal sections. See text for details. (b) Superior, inferior and lateral limits of condylar head structure. See text for details.

**Figure 3:**
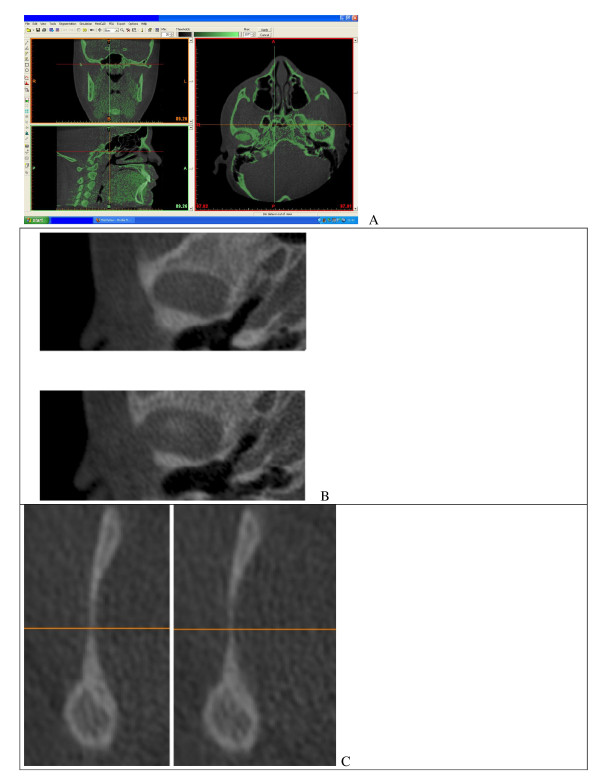
**(a-c) Illustration on the procedure for condylar 3-D reconstruction**. (a) Firstly, a mask which includes all facial bone structures is created with the Mimics software; the proper threshold is selected to individuate TMJ area (26-3071). (b) The superior limit of condylar head is selected when the first white area appears in the upper articular region, while scrolling the images from the upper to the lower regions of the joint space. (c) The inferior limit is selected when the sigmoid area disappears.

Specifically, after enlargement of the TMJ area, the remaining surrounding structures were progressively removed using various sculpting tools for the upper, lower, and side condylar contours (cortical bone), as shown in Figure 1. The segmentation was made on coronal views, and the superior, inferior, and lateral limits of the condyle were standardized (Table 1 and Figure 2a-b). On the coronal views, the superior contour of the condyle was defined where the first radiopaque point was viewed in the image depicting the synovia; the lateral contours for each section were easily identified through clear visualization of the cortical bone (Figure 2a). The inferior contour of the condyle was traced where its section passed from an "elipsoidal" shape (owing to the presence of anterior crest of the condylar head) to a more "circular" shape (suggesting that the view was at the level of the condylar neck) (Figure 2b). Accordingly, the condyle CBCT data sets were segmented with a dedicated Mimics™ tool to construct a new mask that included only the mandibular condyle (Figures 1 and 3). After the condylar segmentation, 3-D multiplanar reconstructions were produced (Figure 1), and volumetric (mm^3^) and surface measurements (mm^2^) were made for each condyle through the Mimics™ automatic function (Figure 1)

**Table 1:** Definitions of Anatomic Contours

Landmark definition	Definition
Superior contour (UCo)	Most superior extent of the mandibular condyle from the anterior, mesial, lateral, and superior planes of view.

Lateral contours (LCos)	Most lateral extent of the mandibular condyle viewed from the anterior, posterior, lateral, and superior planes of view

Inferior contour (ICo)	Most inferior extent of the mandibular condyle from the anterior, mesial, lateral, and superior planes of view. The exact cut where the surface of coronal section increase (because of the beginning of sigmoid area) instead of decrease. This cut allowed the segmentation of the condylar head, without the sigmoid area.

### Studies on method error

To assess the intra-operator error, the CBCT data of 10 patients were processed by the same operator (S.T.) twice (one week apart [11]) and the differences in the condylar volumes and condylar surfaces were evaluated using the Wilcoxon Signed Rank test, because of the small number of subjects included in the study of intra-operator method error. The intra-operator method error was expressed with the formula:

$$\text{E}=\surd \Sigma {\left(\text{x1}-\text{x2}\right)}^{\text{2}}/\text{2n}$$

Where X1 and X2 are the two measurements and n is the number of double measurements.

### Statistical analyses

The volume and surface measurements were processed and analyzed using SPSS 14.0 (SPSS Inc, Rainbow Technologies, Chicago, Ill). For the three variables, the mean, standard deviation, range, minimum, maximum, 95% confidence interval for mean and the results for normality distribution (Kolmorgorov-Smirnov Z test) were calculated, based on gender and TMJ side (Table 2 and Table 3).

**Table 2:** Descriptives statistic

		TMJ side				Gender			
		**Condylar volume (mm^3^)**		**Condylar surface (mm^2^)**		**Condylar volume (mm^3^)**		**Condylar surface (mm^2^)**	

		**RIGHT** **(N = 150 TMJ)**	**LEFT** **(N = 150 TMJ)**	**RIGHT** **(N = 150 TMJ)**	**LEFT** **(N = 150 TMJ)**	**MALES** **(N = 148 TMJ)**	**FEMALES** **(N = 152 TMJ)**	**FEMALES** **(N = 152 TMJ)**	**MALES** **(N = 148 TMJ)**

**Mean**		693.61	666.99	411.24	389.41	691.26	669.65	394.77	406.02

**SD**		62.82	48.67	57.99	56.63	54.52	58.80	60.73	55.22

**95% Confidence interval for mean**	**Lower Bound**	683.48	659.15	401.88	380.27	682.4	660.22	385.04	397.05

	**Upper Bound**	703.75	674.85	420.59	398.55	700.1	679.07	404.5	414.99

**Minimum**		487.5	560.4	310.2	310.2	560.4	487.5	310.2	311.6

**Maximum**		871.2	798.4	498.8	498.7	798.4	871.2	498.8	498.7

**Range**		383.7	238	188.6	188.5	238	383.7	188.6	187.1

**Kolmogorov-Smirnov Z**		0.064	0.062	0.055	0.058	0.061	0.051	0.063	0.052

**p**		0.2	0.2	0.2	0.2	0.2	0.2	0.2	0.2

**Table 3:** Descriptive statistic for Morphological Index (MI)

	N	Mean	Std. Deviation	Range	Minimum	Maximum	95% Confidence Interval for mean		Kolmogorov-Smirnov Z	p
							Lower bound	Upper bound		
**Morphological Index (females)**	152 TMJ	1.72	0.19	1	1.15	2.15	1.69	1.75	0.61	0.200
**Morphological Index (males)**	148 TMJ	1.72	0.14	0.69	1.43	2.12	1.69	1.74	0.51	0.200
**Morphological Index (right)**	150 TMJ	1.70	0.16	0.88	1.15	2.03	1.68	1.73	0.58	0.200
**Morphological Index (left)**	150 TMJ	1.73	0.18	0.87	1.28	2.15	1.71	1.76	0.55	0.200
**Morphological Index (whole sample)**	300 TMJ	1.72	0.17	1	1.15	2.15	1.70	1.74	0.44	0.200

As all the data were normally distributed (Table 2), the *t*-test for independent samples was employed to calculate the statistically significant differences between the males and the females (Table 4) and the paired samples t-test to calculate the statistically significant differences between the right and left sides (Table 4).

**Table 4:** Results of the test t for independent samples and the paired samples t-test applied at the data about condylar volume, condylar surface and Morphological Index

CONDYLAR VOLUME						
	**RIGHT**	**LEFT**	**Mean difference**	**95% confidence interval of difference**	**t**	**Sig**.

**Mean**	693.61	666.99	25.39	Lower: 13.80	4.33	0.000 **
**SD**	62.82	48.67	5.86	Upper: 36.98		

	**MALES**	**FEMALES**	**Mean difference**	**95% confidence interval of difference**	**t**	**Sig**.

**Mean**	691.26	669.65	21.61	Lower: 8.72	3.29	0.001 **
**SD**	54.52	58.80	6.55	Upper: 34.5		

**CONDYLAR SURFACE**						

	**RIGHT**	**LEFT**	**Mean difference**	**95% confidence interval of difference**	**t**	**Sig**.

**Mean**	411.24	389.41	17.15	Lower: 4.41	2.66	0.009**
**SD**	57.99	56.63	6.45	Upper: 29.89		

	**MALES**	**FEMALES**	**Mean difference**	**95% confidence interval of difference**	**t**	**Sig**.

**Mean**	406.02	394.77	11.25	Lower: 1.95	1.68	0.095 NS
**SD**	55.22	60.73	6.70	Upper: 24.45		

**MORPHOLOGICAL INDEX**						

	**RIGHT**	**LEFT**	**Mean difference**	**95% confidence interval of difference**	**t**	**Sig**.

**Mean**	1.70	1.73	-0.03	Lower: -0.069	-1.57	0.118 NS
**SD**	0.16	0.18	0.019	Upper: 0.0078		

	**MALES**	**FEMALES**	**Mean difference**	**95% confidence interval of difference**	**t**	**Sig**.

Mean	1.72	1.72	0.018	Lower: -0.03	0.09	0.92 NS
SD	0.14	0.19	0.019	Upper: 0.04		

Finally, a multiple regression analysis using condylar volume as the dependent variable was performed to assess the influence of side, gender, and condylar surface, as a multiple factor. For each test P was set at 0.05 value.

## Results

No significant difference was found between the two measurements made by the same operator, for the volume (*Z *= -0.204; *p *= 0.838) or for the surface (*Z *= -1.329; *p *= 0.184), as assessed with the Wilcoxon Signed Rank test.

The mean differences between the first and the second measurements are -1.05 mm^2 ^and -2.71 mm^3^, with a method error of 2.35 for the surface and 6.06 for the volume.

The results of the paired samples *t*-test, employed to calculate the statistically significant differences between the right and left sides, showed that the right condyle shows a significantly higher surface than the left condyle (Table 4).

The results of the *t*-test for independent samples to assess the differences between the males and females were that Males show a higher condylar surface when compared with that of females, without statistical significance (Table 4).

For the condylar volume, the differences in the condylar volume between the right and the left sides and those between males and females are statistically significant (Table 4). The right condyle shows a significantly higher volume than the left condyle, and males show a higher condylar volume when compared with females (Table 4). Table 4 also shows the results of MI and the comparisons between males and females as well as those between the right and left sides. No significant difference is seen in the MI between the genders and the sides of TMJ.

Multiple regression analysis demonstrates that the dependent variable (condylar volume) is significantly influenced by side, gender, and condylar surface, as multiple factor; each factor appears to affect the TMJ volume at a significant level.

## Discussion

To the authors' knowledge and until the advent of CBCT as an analysis, the condylar volume and surface have not been routinely calculated, although a recent study in rats shows enlargement of the left mandibular condyle compared to the rights due to the animal closing to one side, [12] thus suggesting that volumetric data can be useful in understanding the TMJ function.

Using semiquantitative reverse-transcription polymerase chain reaction, a study examined the expression of insulin-like growth factor-1, fibroblast growth factor-2, and their receptors in the mandibular condylar cartilage of 28 day-old rats at 3, 7, and 14 days after placing intraoral appliances designed to produce a lateral functional shift of the mandible [13]. This shift resulted in a transverse rotation of the mandible so that the condyle on the side away from the shift was distracted anteriorly (ie, protruded) from the glenoid fossa, while the contralateral condyle remained in place or moved slightly posteriorly (ie, nonprotruded). Gene expression for genes resulted significantly different between the protruded and the nonprotruded condylar sides. More specifically, mRNA expression on the protruded side compared with age-matched controls was altered in the opposite direction from the nonprotruded side, generating alterations in proliferative activity of cartilage.

According to the view that growth of the mandibular condylar cartilage adapts to its local functional-biomechanical environment, cartilage thickness was also investigated, [14] and it resulted on the protruded side significantly greater than that in the controls at each time interval, with the difference increasing slightly with time. Trends on the nonprotruded side were generally opposite, culminating in reduced thickness and proliferation after 14 days, suggesting that changes in mandibular condylar cartilage thickness and proliferative activity might accompany a lateral functional shift of the mandible also in growing subjects, so generating a different condylar volume.

A certain role of the trigeminal motor nucleus in the postnatal ontogeny of the mammalian craniofacial skeleton was also demonstrated in a study where a group of 42 adult rats underwent stereotaxic surgery: [15] among them, 21 received electrolytic lesions to their left-side nucleus (lesioned group) while 21 received a stimulation with no actual electrolytic lesion (sham group). The masticatory muscle weight, and osteological growth were compared: lesioned animals showed significant differences from sham animals, but, interestingly, sham animals also demonstrated significant between-side differences for medial pterygoid muscle weight, mandibular height and length data, suggesting that even relatively slight damage to trigeminal motor nucleus can create morphological changes within the craniofacial complex, mostly with the mandible.

Studies on the condylar development [16] stated that the mandibular condyle is convex along the surface that receives the force, wider in the medio-lateral dimension, and has an oval shape antero-posteriorly. This observation and others, such as the relation of the articular disc with the condyle and the temporal bone, muscle attachments, and occlusion, requires a 3-D approach to analyze the TMJ complex [16]. Matrix and cell surface proteoglycans (PGs) may play important roles in the control of cellular actions of heparan-binding growth factors such as fibroblast growth factor (FGF) during chondrogenesis and osteogenesis, as the inhibition of PG synthesis resulted in reduced incorporation of chondroitin sulfate into cartilage and bone matrix, that was associated with a 75% reduction in cell growth in condyle (equivalent to a reduced volume), determined by DNA synthesis, and in collagenous matrix synthesis in condyle, evaluated by immunohistochemistry. Morphological and quantitative autoradiographic analyses also showed that inhibition of PG synthesis blocks bone matrix formation by perichondral progenitor cells in condyles.

The purpose of our study has not been to test the accuracy of linear measurements calculated with CBCT scans, but to utilize the proven accuracy [17,18] for measuring condylar volume and surface in a selected Caucasian young adult population with malocclusion and no pain or dysfunction of TMJ, to improve the knowledge of condylar shape in subjects with malocclusion. Although condylar volume and condylar surface are not commonly and presently evaluated in clinics, 3D diagnostic tools should become more prevalent in the future.

The study on method errors indicates that CBCT allows for accurate volumetric and surface evaluation of mandibular condyle. No significant difference was found between the two measurements made by the same operator.

In this study, the data of only young adult subjects were included. Older subjects were excluded because they are expected to suffer more frequently from progressive degenerative bone changes owing to the development of TMJ osteoarthritis than do the younger patients [3].

Furthermore, Schluetera et al. (2008) [19] developed a reliable and reproducible method for cutting the condylar area and calculating its volume. However, they used different software and cut the condyle at the base of its neck, while the authors believe that the data on the isolated condylar head can be better associated with the growing mandibular process.

### Sexual dimorphism

The mean difference between the means of condylar volume in males and females is 21.61 mm^3^, which is about the 3.3% of the mean condylar volume in the whole sample. This difference was statistically significant (Table 4).

The difference between the means of condylar surface in males and females is 11.25 mm^2^, which is about 2.8% the condylar surface of the whole sample), but not statistically significant (Table 4).

These differences expressed as percentages respect to the means found in the whole sample, are in accordance with those of a recent study that investigated the female-to-male proportions in head and facial linear dimensions, and found a mean difference of 3-5% in the frontal and lateral views in young and adult patients, between males and females [20].

The differences in volume or surface suggest high variability among the subjects. But, it is not clear the clinical relevance, as no subject included in this report had any pain or dysfunction of TMJ, but only malocclusions.

For the variable MI that indicates the ratio between volume and surface, the difference between females and males is 0.018, which corresponds to about 1% of the mean MI in the whole sample. There is not a statistically significant difference between males and females in the MI values (Table 4).

These data confirm that sexual dimorphism concerns mostly with condylar volume, rather than with condylar surface or the Morphological Index, almost in subjects with malocclusions.

### Difference between the right and left sides

The mean difference between the means of condylar volume in the right and the left TMJ is 25.39 mm^3^, which is about the 3.9% of the mean condylar volume in the whole sample (*p *< 0.01)

The difference between the means of condylar surface in the right and the left TMJ is 17.15 mm^2^, which is about 5.45% the condylar surface of the whole sample (*p *< 0.01).

The clinical relevance of this high variability in the mean differences is yet to be understood, considering that no subject included in the sample had any pain or dysfunction of TMD, but only malocclusions. Consequently, the observed anatomical differences between the two condyles cannot be related to the subject's predisposition to develop any specific pathology on one side, rather than on the other. However, these types of associations are better evaluated through longitudinal studies.

In conclusion, it is hard to confirm whether the dimensions measured on the images correspond to the real dimensions of the structures, because the data obtained here cannot be compared with the anatomical truth. Nevertheless, it is attempted to interpret these data on the basis of the asymmetry that is normal to all human-body structures.

A comparison between the results of this study and those of a recent study (based on 30 subjects with class I malocclusion [aged 15-30 years]), reveals differences, though not statistically significant, in the size of the mandibular condyle between the right and left sides: [21] mean of medio-lateral condylar diameter is higher on the right side than that on the left side. However, the contrary is true for the antero-posterior condylar diameter.

Besides, other variables concerning TMJ morphology also show significant difference between the two sides. The depths of the mandibular fossae and the mean anterior and posterior joint spaces are higher for the right TMJs than those of the left TMJs; however, the contrary is true for the mean superior joint space. As regards the posterior joint space, the difference between the two sides is significant. These observations corroborate the present finding of a physiologically significant asymmetry of condylar size, in almost all subjects with malocclusion.

Also, in a study conducted on eight cadaveric heads (16 dissected mandibular condyles) to investigate the position of auriculo-temporal nerve in relation to the mandibular condyle, the nerve was observed to have a closed anatomic relationship with the condyle; however, although it was in direct contact with the condylar neck in all the cases, there was no correlation between the positions of the nerves in the right and left sides, suggesting marked asymmetry in the right and left condylar anatomy [22].

The asymmetry observed in the present study between the right and left sides can also be related to the presence of a preference side for mastication in subjects with malocclusion.

According to literature, the most significant morphologic alterations and positioning asymmetries of TMJ structures are related to the absence of teeth, dental abrasion, premature occlusal contact points, functional mandibular deviations, unilateral posterior crossbites, and dentoskeletal asymmetries. Specifically, the rule of articular cartilage - a growth center - has been demonstrated to respond to the degenerative changes and nonphysiological strain in the joint areas (application of soft diet or extractions), through changes in the thicknesses of single cartilage layers and total layer thickness, causing a change in the vertical dimensions and width, which is manifested by changes in the maturation processes of centrally unloaded cartilage sections in rats [23]. With regard to the condylar volume, an earlier study demonstrated that hyperplasia of mandibular condyle is histologically characterized by the presence of an uninterrupted layer of undifferentiated germinative mesenchymal cells, a layer of hypertrophic cartilage, and the presence of islands of chondrocytes in the subchondral trabecular bone [24]. The present study suggests that about 4-6% of difference in volume and surface can be observed between the two sides of subjects with malocclusion and without pain or dysfunction in TMJs, along with a wide range of percentages in physiological asymmetry. It has also been shown that the shape and linear dimensions of the mandibular condyle are highly variable [25].

Thus, in the light of the wide range of percentages of asymmetry between the right and left sides, these clinical variables do not seem to be accurate or clinically useful to determine the existence of an abnormal condition in a subject with malocclusion and without pain of dysfunction in TMJs.

Instead, MI could be a better indicator, because no statistically significant difference is found in the MI values between the right and the left sides, or between males and females, in young adult Caucasian subjects with malocclusion and without pain or dysfunction in TMJs.

In young adult Caucasian subjects with malocclusion and without pain or dysfunction in TMJs, its range of values seem to be (both in males and females; both in the right and left sides) 1.72 ± 0.17 mm, with a total range from 1.15 to 2.15 mm.

One can interpret the data on volume and surface of condyle from a clinical point of view. But, before doing so the clinician must know that (i) the functional loads applied to the TMJ might influence TMJ's morphology, (ii) the shape and function are intimately related, although this concept is given due importance only in studies on class II and class III skeletal patterns, [26] and (iii) both the volume and the surface of a condyle differ between the right and left sides, in subjects with malocclusion and without pain or dysfunction in TMJs.

With the advent of 3-D CBCT scan, the clinician can request the radiologist to directly evaluate or calculate condylar volume and surface, as also the MI, using dedicated software.

If the standards of MI are established, then the clinician can use them to directly establish the presence or absence of an abnormal shape.

It would also be useful to evaluate the abnormality in the TMJ morphology of a specific patient, based mostly on the fact that no difference exists in the MI score between males and females or between right and left TMJs of almost all Caucasian young adult subjects, suggesting that it must be between 1.15 and 2.15 in subjects with malocclusion and TMJ without pain or dysfunction.

### Limits of the study

Numerous factors should be considered in applying the results of this investigation to clinical situations. The 3-D volumetric depiction depends on the appropriateness of segmentation, the thresholding of bone pixel values, and the accurate suppression of the surrounding tissue values to enhance the structure of interest. The depiction is dependent on the software algorithm, the spatial and contrast resolution of the scan, the thickness and degree of calcification or cortication of bone structure, and the technical skill of the operator. The Mimics software used in this study enables semi-manual segmentation by interaction of the operator with the data to produce a visually acceptable 3-D rendering. According to Periago (2008), [27] these limitations cause deficiencies or voids in the surface of the image, which occur in regions represented by few voxels or have gray values still representing the bone, but outside the threshold. These areas include the cortical bone of the mandibular condyle, and thus may lead to greater identification error (e.g., for condylar contours) and consequently to measurement error. However, no significant difference is found between the intra-observer method errors, thus suggesting that an accurate procedure of segmentation can occurr.

## Conclusion

This study in young adult Caucasian subjects with malocclusion without pain of dysfunction in TMJs shows significant variability in condylar volume and surface between both the genders and the two sides of the mandible. In particular, the MI of the mandibular condyle (volume/surface) is 1.72 ± 0.17 in this sample, with no significant difference between both genders and right and left sides. This information can prove clinically useful in establishing the diagnostic criteria for condylar volume and surface is subjects with malocclusion and without pain and dysfunction of TMJ.

Furthermore, the generation of stable and repeatable data on condylar volume and surface in truly functionally normal joints (without TMJ pain or dysfunction, and malocclusion), obtained in multi-center studies, could clarify the relationship between the diagnosis of TMJ dysfunction and the measurements of condylar volume and surface.

## Competing interests

The authors declare that they have no competing interests.

## Authors' contributions

ST, the principal investigator, conceived the study, carried out the protocol, partecipated in the recording of data, performed the statistical analysis, the interpretation of data, and partecipated to the coordination of the authors. FF cordinated the other authors. The other authors partecipated in the collecting of data. ST wrote the manuscript, that was approved by all the other authors.

## Pre-publication history

The pre-publication history for this paper can be accessed here:

http://www.biomedcentral.com/1471-2342/10/28/prepub
